# Mesenteric occlusive disease of the inferior mesenteric artery is associated with anastomotic leak after left-sided colon and rectal cancer surgery: a retrospective cohort study

**DOI:** 10.1007/s00384-021-04089-0

**Published:** 2022-01-07

**Authors:** Melissa N. N. Arron, Richard P. G. ten Broek, Carleen M. E. M. Adriaansens, Stijn Bluiminck, Bob J. van Wely, Floris T. J. Ferenschild, Henk F. M. Smits, Harry van Goor, Johannes H. W. de Wilt, André S. van Petersen

**Affiliations:** 1 Department of Surgery, Radboud Institute for Health Sciences, Radboud University Medical Center, Geert Grooteplein Zuid 10 (internal post 618), 6525 GA Nijmegen, The Netherlands; 2 Department of Surgery, Bernhoven Hospital, Uden, The Netherlands; 3 Department of Surgery, Maashospital Pantein, Boxmeer, The Netherlands; 4 Department of Radiology, Bernhoven Hospital, Uden, The Netherlands

**Keywords:** Mesenteric occlusive disease, Colorectal cancer, Colorectal surgery, Anastomotic leakage

## Abstract

**Purpose:**

Anastomotic leak (AL) is a serious complication following colorectal surgery. Atherosclerosis causes inadequate anastomotic perfusion and is suggested to be a risk factor for AL. The aim of this study was to investigate the association of mesenteric occlusive disease on preoperative computed tomography (CT) scan with AL after left-sided colon or rectal cancer surgery.

**Methods:**

This was a retrospective, multicenter cohort study including 1273 patients that underwent left-sided or rectal cancer resection between 2009 and 2018 from three hospitals in the Netherlands. AL patients were 1:1 matched with non-leak patients and preoperative contrast-enhanced CT-scans were retrospectively analyzed for mesenteric atherosclerotic lesions. The main outcome measure was the presence of mesenteric occlusive disease on the preoperative CT-scan.

**Results:**

Anastomotic leak developed in 6% of 1273 patients (*N* = 76). Low anterior resection and stage I–III disease were statistically significant associated with AL (*p* = 0.01, *p* = 0.04). No other statistically significant differences in patient characteristics between AL and non-leak patients were found. A clinically significant stenosis (≥ 70–100%) of the inferior mesenteric artery was statistically significant more frequent present in AL patients, compared to non-leak patients (*p* < 0.01). No statistically significant differences in the presence of mesenteric occlusive disease of the celiac artery and superior mesenteric artery between AL patients and non-leak patients were found.

**Conclusion:**

Mesenteric occlusive disease of the IMA on preoperative CT-scan is associated with AL after left-sided colon or rectal resection for cancer. Preoperative identification of high-risk patients with a preoperative CT-scan of the mesenteric vasculature might be useful to reduce the risk of AL.

**Supplementary Information:**

The online version contains supplementary material available at 10.1007/s00384-021-04089-0.

## Introduction

Anastomotic leakage (AL) is a severe complication of colorectal cancer surgery. It is associated with severe morbidity and high mortality rates [1–3]. Despite extensive research, incidence of AL remains high, with distal anastomoses being associated with the highest leak rates [4]. Many risk factors for AL have been identified, including smoking and male sex [2, 5, 6]. Inadequate anastomotic vascular perfusion has also been suggested to be an important contributor to the development of AL [7, 8].

Perfusion is essential in wound healing in general, and inadequate anastomotic perfusion might therefore threaten healing of anastomotic tissue [7, 9]. Atherosclerotic calcification is one of the main causes of inadequate perfusion and is also suggested to be a potential risk factor for AL [10, 11].

Several recent studies demonstrated that atherosclerotic calcification of the aorta-iliac tract on preoperative CT-scan is associated with an increased risk of AL [12–14]. Although calcification in large arteries might mirror the anastomotic perfusion, the blood supply of the colorectal region is primary provided by the inferior mesenteric artery (IMA). In addition, adequate anastomotic perfusion also depends on the presence and patency of the collateral circulation (e.g. Riolan) as well as on the compensatory function of the superior mesenteric artery (SMA) and celiac artery (CA) [15]. Until now, evidence on the role of mesenteric occlusive disease in AL is limited and only one small study has been performed yet [16].

Preoperative identification of patients at risk of inadequate anastomotic perfusion with a preoperative CT-scan of the mesenteric vasculature is low-cost and minimally invasive. It may provide opportunities to modify these vascular risk factors and to take preventive measures (e.g. construction of a diverting stoma) to reduce the risk for AL. We hypothesize that mesenteric occlusive disease of the IMA, SMA, and CA, diagnosed with preoperative abdominal CT-scan is associated with AL. Therefore, in the present study we investigated the association of mesenteric occlusive disease on preoperative abdominal CT-scan with AL after left-sided colon or rectal cancer surgery in a population-based cohort.

## Materials and methods

All patients (*N* = 1273) who underwent elective or emergency colorectal resection with construction of an anastomosis for primary left-sided colon, sigmoid or rectal cancer at three Dutch hospitals, Radboud University Medical Center, Bernhoven Hospital and Maas hospital Pantein, between January 2009 and December 2018 were retrospectively analyzed. Data on baseline and surgery-related parameters was retrieved from local extractions of the Dutch ColoRectal Audit (DCRA), a population-based audit. The following baseline and surgery-related parameters were retrieved: age, body mass index (BMI), ASA score, comorbidities, pathological TNM stage, surgical procedure, (deviating) stoma creation, AL, surgical treatment for AL, stoma construction at reoperation for AL, intensive care unit (ICU) admission, 90-day hospital readmission, and length of hospital stay. In this study, the low-tie technique was used in rectal cancer surgery. This is according to previous studies demonstrating that the low-tie technique is associated with a higher blood flow ratio compared to high-tie, which may benefit anastomotic perfusion [8]. More detailed information on AL was collected from the electronic patient records of all AL patients. The following data items were collected: smoking status, date of reintervention for AL, conservative or radiological treatment for AL, and stoma reversal during the postoperative follow-up. Data regarding AL from the electronic patient record was only collected after written informed consent of the patient. Eight AL patients declined to give consent or did not respond, and their data was not included in the analysis.

**Fig. 1:**
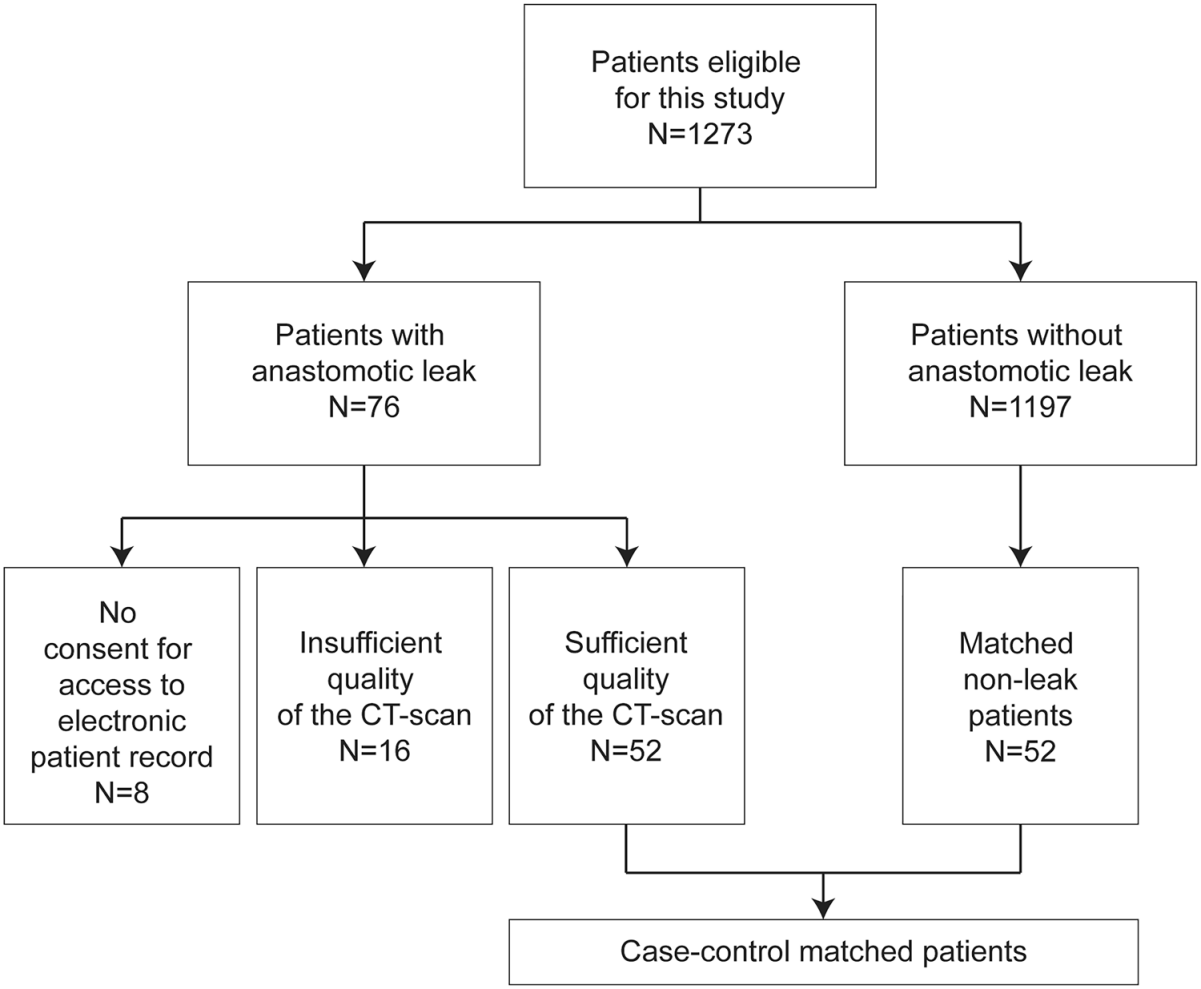
Flowchart

**Fig. 2:**
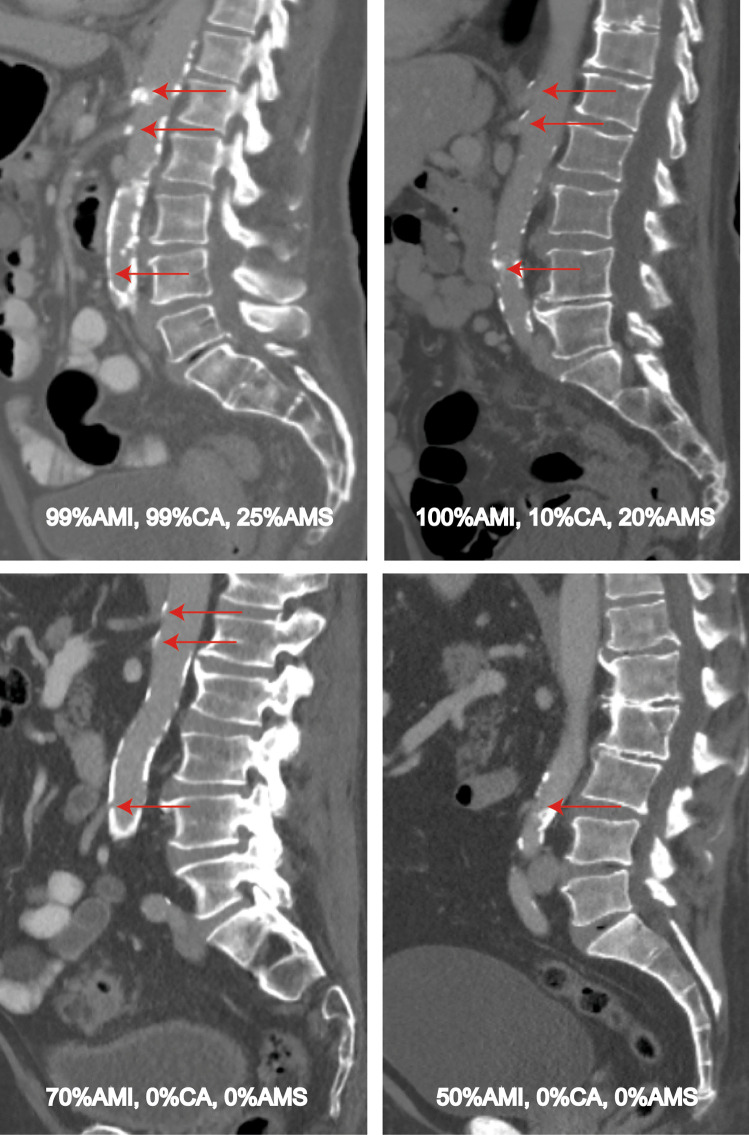
Abdominal CT-images of patients with mesenteric occlusive disease

To assess the presence of mesenteric occlusive disease, preoperative contrast-enhanced CT-scans were retrospectively reviewed for atherosclerotic lesions by a vascular surgeon (A. P.) and radiologist (H.S.). These preoperative contrast-enhanced CT-scans with a 5-mm slice thickness were performed for oncological staging with no specific attention for the presence of atherosclerotic lesions. Of 76 AL patients, CT-scans of 52 patients were available for assessment and 24 CT-scans of AL were not available due to insufficient quality (*N* = 16) or the patient did not give consent for access to their electronic patient record (*N* = 8) (see Flowchart; Fig. 1). We performed case–control matching to create a cohort including AL and non-leak patients with equal risks on the presence of mesenteric occlusive disease on the CT-scan. Patients with AL of whom the CT-scan was available (*N* = 52), were matched with non-leak patients (*N* = 52) selected from the same cohort to create a representative sample of the total population. Non-leak patients were chosen using 1:1 case–control matching with AL patients based on age, BMI, and cardiovascular comorbidity; all risk factors for mesenteric occlusive disease. The patency of the IMA, SMA, and CA was reviewed during the inspiration phase of the CT scan. The degree of stenosis was classified into four groups: no atherosclerotic lesions, stenosis < 50%, stenosis ≥ 50%–70%, and clinically significant stenosis ≥ 70%–100%. Figure 2 shows CT-images of these four groups. The presence of a collateral circulation between the IMA and SMA was graded according to the following classification: 0 — not visible/absent, 1 — present, but poor visibility, 2 — present and good visibility. The reviewers were blinded for clinical information to prevent bias. Discrepancies between the reviewers on the scoring were solved by re-examination of the CT-scan until consensus was reached. The individual scores of both reviewers and the final score (after consensus was reached) are depicted in suppl. Table 1.

The primary outcome of this study was the presence of mesenteric occlusive disease on the preoperative CT-scan. Secondary outcome was colorectal AL, defined as clinically relevant AL that requires reintervention within 90 days after surgery.

The local medical ethical committee reviewed and approved the study protocol (registration number 2019–5269).

## Statistical analysis

The data were analyzed using the SPSS statistical software version 25.0. Differences in baseline characteristics, postoperative outcomes and the presence of stenoses of the mesenteric arteries (no, < 50%, ≥ 50–70%, ≥ 70–100%) between AL patients and non-leak patients were presented as frequencies and percentages and analyzed using Fisher’s exact test (observed count < 10) or chi^2^-test for binominal data. For skewed continuous data (e.g., length of hospital stay) the Mann–Whitney *U* test was used, and data was presented as median with interquartile range (IQR). All reported *p*-values were two-sided, a *p*-level of < 0.05 was used as the level of significance.

## Results

### Baseline characteristics

Between January 2009 and December 2018, a total of 1273 patients underwent colorectal resection with primary anastomosis for colorectal cancer in this cohort. The median age of patients included in this study was 67 years (IQR 61–74). In 905 patients (71%) a left-sided colectomy or sigmoid resection was performed and 367 patients (29%) underwent low anterior resection (LAR). Two hundred patients (16%) received a deviating stoma at resection of the tumor. There were 22 AL (29%) patients with a history of smoking or who were currently smoking. Univariable analysis revealed LAR and stage I–III disease to be risk factors for AL (*p* = 0.01, *p* = 0.04). No other differences in baseline characteristics were found between AL patients and non-leak patients at univariate analysis as depicted in Table 1. In the case–control matched patient cohort, there were also no baseline differences between AL patients and matched non-leak patients, except for LAR and male sex being associated with an increased risk of AL (*p* = 0.04). The median age was 65 years (IQR 57–70), without statistically significant differences between groups. A total of 69 patients underwent left colon resection (66%), 35 patients (34%) underwent rectal resection, and 17 patients (15%) received a deviating stoma at primary resection. Twenty-four patients were diagnosed with any cardiovascular comorbidity (23%), with equal distribution over the AL and matched non-leak group. The results are depicted in Table 1.

**Table 1:** Baseline patient-, tumor-, and treatment characteristics

	**Total population *N* = 1273**	**Unmatched patients**			**Case–control matched patients**		
		**Anastomotic leakage *N* = 76**	**No anastomotic leakage (*N* = 1197)**	*p*-value *AL* vs. *no AL*	**Anastomotic leakage *N* = 52**	**Matched “no anastomotic leak” cohort *N* = 52**	*p*-value *AL* vs. *matched no AL*
	*N* (%)	*N* (%)			*N* (%)		
Age							
< 70	758	53 (69.7)	705 (58.9)	0.07	40 (76.9)	40 (76.9)	1.00
≥ 70	514	23 (30.3)	491 (41.1)		12 (23.1)	12 (23.1)	
BMI							
< 18.5	16	0 (0.0)	16 (1.3)	0.46	0 (0.0)	0 (0.0)	0.91
18.5–25.0	431	30 (39.5)	401 (33.5)		21 (40.4)	19 (36.5)	
25.1–29.9	550	34 (44.7)	516 (43.1)		24 (46.2)	26 (50.0)	
30–34.9	222	10 (13.2)	212 (17.7)		6 (11.5)	6 (11.5)	
Missing	55	2 (2.6)	53 (4.3)		1 (1.9)	1 (1.9)	
ASA							
I	275	16 (21.1)	259 (21.7)	0.48	10 (17.3)	19 (36.5)	0.06
II	766	43 (56.6)	723 (60.5)		30 (57.7)	28 (53.8)	
III +	218	17 (22.4)	201 (16.8)		12 (23.1)	5 (9.6)	
Missing	13	0 (0.0)	13 (1.1)		-	-	
Comorbidity							
Myocard infarction	128	11 (14.5)	117 (9.8)	0.23	5 (9.6)	5 (9.6)	1.00
Peripheral arterial failure/AAA	44	2 (2.6)	42 (3.5)	1.00	1 (1.9)	1 (1.9)	1.00
Cerebrovascular disease (CVA, TIA)	89	6 (7.9)	83 (6.9)	0.65	2 (3.8)	5 (9.6)	0.44
Diabetes mellitus	166	10 (13.2)	156 (13.0)	1.00	6 (11.5)	5 (9.6)	1.00
Pathological tumor stage							
(y)pT0-pT2	554	32 (42.1)	522 (43.6)	0.67	23 (44.2)	13 (25.0)	0.08
(y)pT3-pT4	688	44 (57.9)	644 (53.8)		29 (55.8)	38 (73.1)	
(y)pTX/unknown	30	0 (0.0)	30 (2.6)		-	1 (1.9)	
Pathological node stage							
pN0	704	40 (52.6)	664 (55.5)	0.30	28 (53.8)	31 (59.6)	0.83
pN1	270	21 (27.6)	249 (20.8)		12 (23.1)	13 (25.0)	
pN2	142	6 (7.9)	136 (11.4)		5 (9.6)	8 (15.4)	
pNx/unknown	156	9 (11.8)	147 (12.3)		7 (13.5)	-	
Cancer stage							
Stages I–III	1021	67 (88.2)	954 (79.8)	**0.04**	45 (86.5)	45 (86.5)	0.36
Stage IV	94	1 (1.3)	93 (7.8)		1 (1.9)	4 (7.7)	
Unknown	164	8 (10.5)	156 (12.5)		6 (11.5)	3 (5.8)	
Surgical procedure							
Left hemicolectomy/sigmoid resection	905	43 (56.6)	862 (72.1)	**0.01**	29 (55.8)	40 (76.9)	**0.04**
(Low) anterior resection	367	33 (43.4)	334 (27.9)		23 (44.2)	12 (23.1)	
Stoma construction at primary surgery							
No stoma	1008	61 (80.3)	947 (79.2)	0.87	45 (86.5)	41 (78.8)	0.29
Deviating stoma	200	11 (14.5)	189 (15.8)		6 (11.5)	11 (21.2)	
Missing	64	4 (5.3)	60 (5.0)		1 (1.9)	-	

### Postoperative outcomes

AL developed in 76 out of 1273 patients (6%). Of the 76 AL patients, 33 patients (43%) underwent rectal resection, and 43 patients (57%) underwent colon resection. Median days until reintervention for AL were five days (IQR 3–11). AL was treated with a surgical reintervention in 62 patients (82%). Twelve patients (16%) were treated with radiological intervention and one patient (1%) received conservative treatment (i.e. intravenous antibiotics). Of patients treated with surgical reintervention, 55 patients (89%) received a stoma; 33 patients (60%) were treated with an end colostomy and 22 patients (40%) with a deviating stoma. Most of the AL patients who received a stoma either at primary resection or at reintervention still had a stoma at 1 year postoperative (64%, *N* = 35). Four AL patients died within the first year after surgery (5%). The patients with AL had statistically significant worse postoperative outcomes compared to non-leak patients: a higher ICU admission rate (47% versus 12%, *p* = 0.00) and a higher 90-day readmission rate (36% versus 8%, *p* = 0.00). Median length of hospital stay for AL patients was significant longer for AL patients (14 days, IQR 6–23), compared to non-leak patients (5 days, IQR 3–8; *p* < 0.01).

### Assessment of CT-scans for mesenteric occlusive disease

In total, the CT-scans of 52 AL patients and 52 matched non-leak patients were reviewed (Table 2). Sixty-seven patients (64%) had no atherosclerotic lesions of the IMA. Thirty-four of these patients (65% of 52 AL patients) developed AL and 33 (64%) patients did not develop AL. Fifteen patients (14%) had a stenosis < 50% of the IMA, including two AL patients (4%) and thirteen non-leak patients (25%). In ten patients (10%) a stenosis ≥ 50%–70% of the IMA was found, with five patients (10%) in both the AL and non-leak group. A clinically significant stenosis (≥ 70–100%) of the IMA was found in twelve patients (12%), eleven of these patients (21%) developed AL and one patient (2%) did not develop AL (*p* = 0.01). The presence of mesenteric occlusive disease of the IMA was statistically significant associated with AL (*p* = 0.01). Six out of eleven of the AL patients with a clinically significant stenosis of the IMA were diagnosed with a left-sided colon tumor and five had a rectum tumor. In none of these patients a high-tie technique was performed. Four out of eleven AL patients with mesenteric occlusive disease were smokers or had a history of smoking, without statistically significant differences in smoking status between AL patients with mesenteric occlusive disease and AL patients without mesenteric occlusive disease (*p* = 0.67). Ninety-one patients (88%) had no atherosclerotic lesions of the SMA. AL was diagnosed in 44 patients (85%) and 47 patients (90%) did not develop AL. In ten patients (10%) a stenosis < 50% of the SMA was found, of which six patients (12%) developed AL and four patients (8%) did not develop AL. One patient had a stenosis ≥ 50%–70% of the SMA, whom developed AL. A clinically significant stenosis ≥ 70%–100% was found in two patients, with one patient in each of the groups. There were no statistically significant differences found in the presence of mesenteric occlusive disease of the SMA between AL patients and non-leak patients (*p* = 0.68). Seventy-four patients (71%) showed no atherosclerotic lesions of the CA on CT-scan, of which 39 patients (75%) developed AL and 35 patients (67%) did not develop AL. Twenty-six patients (25%) had a stenosis < 50% of the CA. AL developed in eleven of these patients (21%) and fifteen patients (29%) did not develop AL. Two patients (2%) had a stenosis of ≥ 50%–70% of the CA, with one patient (2%) in both groups. A clinically significant stenosis (≥ 70–100%) of the CA was found in two patients that were equally divided over the groups. No statistically significant differences were found in the presence of mesenteric occlusive disease of the CA between AL patients and non-leak patients (*p* = 0.84). The presence of a collateral network between the SMA and IMA could only be observed in a minority of the patients due to insufficient quality of the CT-scans. Collateral arteries were found in fourteen AL patients (26%) and in four matched non-leak patients (7%). None of the patients had a pathological Riolan artery.

**Table 2:** Evaluation of mesenteric atherosclerotic lesions of the celiac artery, superior mesenteric artery and inferior mesenteric artery on preoperative abdominal CT-scan

	**Total patients**	**Anastomotic leakage patients**	**Matched “no anastomotic leak” patients**	*p*-value *AL* vs. *matched no AL*
	*N* = 104	*N* = 52	*N* = 52	
**Celiac artery**				
No stenosis	74 (71.2)	39 (75.0)	35 (67.3)	0.84
Stenosis < 50%	26 (25.0)	11 (21.2)	15 (28.8)	
Stenosis ≥ 50%–70%	2 (1.9)	1 (1.9)	1 (1.9)	
Clinical significant stenosis (≥ 70–100% stenosis)	2 (1.9)	1 (1.9)	1 (1.9)	
**Superior mesenteric artery**				
No stenosis	91 (87.5)	44 (84.6)	47 (90.4)	0.68
Stenosis < 50%	10 (9.6)	6 (11.5)	4 (7.7)	
Stenosis ≥ 50%–70%	1 (1.0)	1 (1.9)	0 (0.0)	
Clinical significant stenosis (≥ 70–100% stenosis)	2 (1.9)	1 (1.9)	1 (1.9)	
**Inferior mesenteric artery**				
No stenosis	67 (64.4)	34 (65.4)	33 (63.5)	0.01
Stenosis < 50%	15 (14.4)	2 (3.8)	13 (25.0)	
Stenosis ≥ 50%–70%	10 (9.6)	5 (9.6)	5 (9.6)	
Clinical significant stenosis (≥ 70–100% stenosis)	12 (11.5)	11 (21.2)	1 (1.9)	

## Discussion

Preoperative prediction of the risk for AL is crucial, since mortality and morbidity rates remain high and the pathophysiology of AL is unknown. In this retrospective cohort study of 1273 patients with left-sided colon or rectal cancer, 76 patients were diagnosed with AL. We showed that the presence of mesenteric occlusive disease of the IMA on preoperative CT-scan was significantly associated with AL in left-sided colon or rectal cancer surgery. No association between the presence of mesenteric occlusive disease of the SMA and CA and AL was found. Univariable analysis revealed LAR and stage I–III disease to be associated with an increased risk for AL. No other differences in baseline patient characteristics were found between AL and non-leak patients. AL patients had statistically significant worse postoperative outcomes including a higher ICU admission rate, a higher 90-day readmission rate and a prolonged length of hospital stay.

There is little available evidence on the association of atherosclerotic lesions of mesenteric arteries on anastomotic healing published yet. Until now, there has been only one study analyzing the association of the presence of atherosclerotic lesions of the mesenteric arteries on preoperative CT-scan with AL. Kornmann et al. analyzed 96 patients undergoing left-sided colon or rectal resection and concluded in contrast to our results, that AL was not associated with the presence of atherosclerotic lesions of the IMA [16]. Also, stenoses ≥ 50% of the IMA were found in 21% of the non-leak patients, which is about two times higher compared to our study. These results may be explained by the fact that they did not compare AL patients to a matched non-leak group and by the lack of sufficient power since only fourteen AL patients were analyzed.

We demonstrated that clinically significant stenosis of the IMA is statistically significant more prevalent on the preoperative abdominal CT-scan of AL patients. This finding can be explained by mesenteric occlusive disease limiting the blood flow of the IMA, creating a “functional high-tie” and as a result, limiting anastomotic perfusion and contributing to the development of disrupted healing [8, 17].

In contrast to the limited available evidence on the association of mesenteric occlusive disease with AL, atherosclerotic calcification of larger systemic arteries including the aorta and iliac arteries in relation to AL has been of more interest. Most of these studies quantified atherosclerotic calcification with the calcium score, that is calculated by the total sum of the atherosclerotic load in the analyzed arterial trajectory. Histopathological studies demonstrated that the calcium area is correlated with the atherosclerotic plaque area [18]. Komen et al. analyzed 122 patients (including eleven ALs) undergoing colorectal surgery and concluded that patients with higher calcium scores in both common iliac arteries and the left internal iliac artery were associated with an increased AL risk [14]. Pochhammer et al. found similar results when analyzing 139 patients (including fourteen ALs) who underwent colorectal resection with rectal anastomosis [19]. It is difficult to explain these results anatomically, since the left internal iliac artery and its branches only supply the inferior part of the rectum, and most of the patients in these studies had an anastomosis located in the upper rectum or more proximal. However, in a case–control study of Boersema et al. 145 patients (including 30 ALs) undergoing colorectal resection with left-sided anastomosis, the calcium score did not correlate with the development of AL [20]. Therefore, evidence for the aorto-iliac calcium score as preoperative predictor for AL is conflicting.

To our best knowledge, this is the first study evaluating the association of mesenteric occlusive disease with AL in a large population undergoing left-sided or rectal cancer surgery. Strengths of this study are the significant number of patients with AL that was included and its case–control design. By comparing AL patients to matched non-leak patients from the same cohort, we reduced selection bias and confounding of potential vascular risk factors (e.g. age, BMI, cardiovascular comorbidity). However, data was analyzed, respectively, and we could only evaluate the CT-scans of the patients in whom a CT-scan was performed for preoperative cancer staging. These CT-scans were not primarily performed to assess atherosclerotic lesions, and therefore, some CT-scans were not of sufficient quality to evaluate the mesenteric vascularization. We also could not draw conclusions on the role of the collateral mesenteric circulation due to insufficient quality of the CT-scan. Due to lack of data on smoking status of non-leak patients we did not study the association between smoking status and the presence of mesenteric occlusive disease in the total population. However, there was no statistically significant difference in the smoking status between AL patients with a clinically significant stenosis (≥ 70–100% stenosis) of the IMA and AL patients without a clinically significant stenosis (< 70% stenosis) of the IMA. In addition, smoking is a risk factor for cardiovascular disease and by controlling for cardiovascular disease in the case–control design, we aimed to limit bias of lack on data on smoking status.

In conclusion, we demonstrated that the presence of a clinically significant stenosis of the IMA on preoperative abdominal CT-scan is associated with AL after left-sided colon or rectal resection for cancer. Further prospective studies with additional CT imaging are needed to validate our results and to study the role of collateral vascularization. Preoperative identification of patients at risk of AL with CT-contrast enhanced imaging as part of routine care could be of great value for the current colorectal surgery practice. Until future studies are awaited, additional preoperative imaging such as CT-angiography seems to be indicated in patients with a high risk on AL. When mesenteric occlusive disease of the IMA is diagnosed in these patients, surgeons could perhaps consider preoperative percutaneous transluminal angioplasty (PTA) to improve inadequate anastomotic perfusion to lower the risk of AL.

## Supplementary Information

Below is the link to the electronic supplementary material.

Supplementary file1 (DOCX 17 kb)
