# Supplementary material for: Mesenteric occlusive disease of the inferior mesenteric artery is associated with anastomotic leak after left-sided colon and rectal cancer surgery: a retrospective cohort study
Source: Int J Colorectal Dis. 2022 Jan 7;37(3):631–8. doi: 10.1007/s00384-021-04089-0 (PMC8885551; doi:10.1007/s00384-021-04089-0)
Supplement: Supplementary file 1 — Supplementary file1 (DOCX 17 kb) [file 384_2021_4089_MOESM1_ESM.docx]

| Supplementary table 1. Grading of atherosclerotic lesions on preoperative CT scan by the two examinators and their consensus score | | | | | | | | | |
| --- | --- | --- | --- | --- | --- | --- | --- | --- | --- |
|  | Celiac artery | | | Superior mesenteric artery | | | Inferior mesenteric artery | | |
|  | Examinator 1 | Examinator 2 | Consensus | Examinator 1 | Examinator 2 | Consensus | Examinator 1 | Examinator 2 | Consensus |
| 1 | 0 | 0 | 0 | 0 | 0 | 0 | 0 | 0 | 0 |
| 2 | 0 | 0 | 0 | 0 | 0 | 0 | 0 | 0 | 0 |
| 3 | 0 | 25 | 20 | 0-25 | 25 | 20 | 0 | 25-50 | 20 |
| 4 | 0 | 0 | 0 | 0 | 0 | 0 | 0 | 0 | 0 |
| 5 | 0 | 0-25 | 10 | 0 | 0 | 0 | 0 | 0 | 0 |
| 6 | 0 | 0 | 0 | 0 | 0 | 0 | 0 | 0 | 0 |
| 7 | 0 | 0 | 0 | 0 | 0 | 0 | 0 | 0 | 0 |
| 8 | 0 | 0 | 0 | 0 | 0 | 0 | 50 | 50-75 | 50 |
| 9 | 0 | 0 | 0 | 0 | 0 | 0 | 50 | 50 | 50 |
| 10 | 0-25 | 25 | 25 | 0 | 0 | 0 | 0 | 25 | 20 |
| 11 | 0 | 0 | 0 | 0 | 0 | 0 | 0 | 25-50 | 20 |
| 12 | 0 | 0 | 0 | 0 | 0 | 0 | 0 | 25 | 20 |
| 13 | 50-70 | 50-75 | 70 | 0 | 0 | 0 | 0 | 0 | 0 |
| 14 | 0 | 0 | 0 | 0 | 0 | 0 | 0 | 0 | 0 |
| 15 | 0 | 0-25 | 20 | 0 | 0 | 0 | 0 | 0-25 | 20 |
| 16 | 0 | 0 | 0 | 0 | 0 | 0 | 0-25 | 25-50 | 20 |
| 17 | 0-25 | 0-25 | 25 | 0-25 | 0-25 | 20 | 0-25 | 25-50 | 20 |
| 18 | 0 | 0 | 0 | 0 | 0 | 0 | 0 | 0 | 0 |
| 19 | 0 | 0 | 0 | 0 | 0 | 0 | 0 | 0 | 0 |
| 20 | 0 | 0 | 0 | 0 | 0 | 0 | 0 | 0 | 0 |
| 21 | 0-25 | 0-25 | 20 | 0 | 0 | 0 | 0 | 0 | 0 |
| 22 | 0 | 0 | 0 | 0 | 0 | 0 | 0-25 | 0-25 | 20 |
| 23 | 0 | 0 | 0 | 0 | 0 | 0 | 0 | 0 | 0 |
| 24 | 0 | 0 | 0 | 0 | 0 | 0 | 0 | 0 | 0 |
| 25 | 0-25 | 0-25 | 20 | 0 | 0 | 0 | 0 | 0 | 0 |
| 26 | 0 | 0 | 0 | 10-20 | 25-50 | 25 | 0-25 | 0-25 | 25 |
| 27 | 50 | 50-75 | 50 | 50-70 | 75-90 | 70 | >50 | 25-50 | 90 |
| 28 | 0 | 0 | 0 | 0 | 0 | 0 | 0 | 0 | 0 |
| 29 | 0 | 0 | 0 | 0 | 0 | 0 | 0 | 0 | 0 |
| 30 | 0-25 | 0-25 | 25 | 0 | 0 | 0 | 0 | 0-25 | 0 |
| 31 | 0-25 | 0-25 | 25 | 0 | 0 | 0 | 0-25 | 25-50 | 25 |
| 32 | 10-20 | 0-25 | 25 | 10 | 0-25 | 25 | 0-25 | 25-50 | 25 |
| 33 | 0 | 0 | 0 | 0 | 0 | 0 | 0-25 | 0-25 | 25 |
| 34 | 0 | 25 | 25 | 0 | 0 | 0 | 0 | 0 | 0 |
| 35 | 0 | 0 | 0 | 0 | 0 | 0 | 0 | 0 | 0 |
| 36 | 0 | 0 | 0 | 0 | 0 | 0 | 0 | 0 | 0 |
| 37 | 0 | 0 | 0 | 0 | 0 | 0 | 0 | 0 | 0 |
| 38 | 0 | 0 | 0 | 0 | 0 | 0 | 0 | 0 | 0 |
| 39 | 0 | 0 | 0 | 0 | 0 | 0 | 0 | 0 | 0 |
| 40 | 0 | 0 | 0 | 0 | 0 | 0 | 0 | 0 | 0 |
| 41 | 0 | 0 | 0 | 0 | 0 | 0 | 0-25 | 25-50 | 50 |
| 42 | 0 | 0 | 0 | 0 | 0 | 0 | 0 | 0 | 0 |
| 43 | 0 | 0 | 0 | 0 | 0 | 0 | 0 | 0 | 0 |
| 44 | 10-20 | 50 | 25 | 0 | 0 | 0 | 0 | 0 | 0 |
| 45 | 0 | 0 | 0 | 0 | 0 | 0 | 0 | 0 | 0 |
| 46 | 25 | 20 | 25 | 0 | 0 | 0 | 0 | 0 | 0 |
| 47 | 0 | 0 | 0 | 0 | 0 | 0 | 20 | 20 | 20 |
| 48 | 10 | 10-25 | 25 | 0 | 0-25 | 20 | 0 | 0-25 | 25 |
| 49 | 0-25 | 0-25 | 25 | 0 | 0 | 0 | 0 | 0 | 0 |
| 50 | 0 | 0 | 0 | 0 | 0 | 0 | 0-25 | 50-75 | 50 |
| 51 | 0 | 0 | 0 | 0 | 0 | 0 | 0 | 0 | 0 |
| 52 | 0-25 | 0-25 | 25 | 0 | 0 | 0 | 0 | 25-75 | 50 |
| 53 | 0 | 0 | 0 | 75 | 75 | 75 | 0 | 0 | 0 |
| 54 | 0 | 0 | 0 | 0 | 0 | 0 | 0 | 0 | 0 |
| 55 | 0 | 0 | 0 | 0 | 0 | 0 | 0 | 0 | 0 |
| 56 | 0 | 0 | 0 | 0 | 0 | 0 | 0 | 0 | 0 |
| 57 | 0 | 0 | 0 | 0 | 0 | 0 | 0 | 0 | 0 |
| 58 | 0 | 0 | 0 | 0 | 0 | 0 | 0 | 0 | 0 |
| 59 | 0 | 0 | 0 | 20 | 30 | 25 | 0 | 0 | 0 |
| 60 | 20 | 25 | 25 | 0 | 0 | 0 | 0 | 0 | 0 |
| 61 | 0 | 0 | 0 | 0 | 0 | 0 | 0 | 0 | 0 |
| 62 | 0 | 0 | 0 | 0 | 0 | 0 | 0 | 0 | 0 |
| 63 | 0 | 0 | 0 | 0 | 0 | 0 | 0 | 0 | 0 |
| 64 | 50 | 50 | 50 | 0 | 0 | 0 | 0 | 0 | 0 |
| 65 | 10 | 0 | 10 | 50 | 50 | 50 | 100 | 100 | 100 |
| 66 | 0 | 0 | 0 | 0 | 0 | 0 | 0 | 0 | 0 |
| 67 | 0 | 0 | 0 | 0 | 0 | 0 | 0 | 0 | 0 |
| 68 | 0 | 0 | 0 | 0 | 0 | 0 | 0 | 0 | 0 |
| 69 | 10 | 20 | 20 | 0 | 0 | 0 | 0 | 0 | 0 |
| 70 | 0 | 0 | 0 | 0 | 0 | 0 | 0 | 0 | 0 |
| 71 | 10 | 0 | 10 | 0 | 0 | 0 | 0 | 0 | 0 |
| 72 | 10 | 25 | 20 | 10 | 25 | 15 | 90 | 75 | 90 |
| 73 | 0 | 0 | 0 | 0 | 0 | 0 | 0 | 0 | 0 |
| 74 | 0 | 0 | 0 | 0 | 0 | 0 | 0 | 0 | 0 |
| 75 | 0 | 0 | 0 | 20 | 25-50 | 20 | 25 | 25-50 | 50 |
| 76 | 0 | 0 | 0 | 0 | 0 | 0 | 0 | 0 | 0 |
| 77 | 0 | 0 | 0 | 0 | 0 | 0 | 25-50 | 50 | 50 |
| 78 | 0 | 0 | 0 | 0 | 0 | 0 | 25-50 | 50 | 50 |
| 79 | 0 | 0 | 0 | 0 | 0 | 0 | 0 | 0 | 0 |
| 80 | 0 |  | 0 | 0 | 0 | 0 | 0 |  | 0 |
| 81 | 0 | 0 | 0 | 0 | 0 | 0 | 0 | 0 | 0 |
| 82 | 0 | 25 | 20 | 0 | 0 | 0 | 0 | <50 | 0 |
| 83 | 0 | 0 | 0 | 0 | 0 | 0 | 0 | 50 | 0 |
| 84 | 0 | 0 | 0 | 0 | 0 | 0 | 0 | 0 | 0 |
| 85 | 0 | 0 | 0 | 0 | 0 | 0 | 0 | 0 | 0 |
| 86 | 0 | 0 | 0 | 0 | 0 | 0 | 0 | 0 | 0 |
| 87 | 0 | 0 | 0 | 0 | 0 | 0 | 0 | 0 | 0 |
| 88 | 0 | 25 | 20 | 0 | 0 | 0 | 20 | 25 | 20 |
| 89 | 0 | 0 | 0 | 0 | 0 | 0 | 0 | 0 | 0 |
| 90 | 0 | 25 | 20 | 0 | 0 | 0 | 25 | 25-50 | 25 |
| 91 | 0 | 0 | 0 | 0 | 0 | 0 | 100 | 100 | 100 |
| 92 | 0 | 0 | 0 | 0 | 0 | 0 | 100 | 100 | 100 |
| 93 | 0 | 0 | 0 | 0 | 0 | 0 | 0 | 0 | 0 |
| 94 | 90-100 | 75-99 | 99 | 0-25 | 25 | 25 | 90 | 75-99 | 99 |
| 95 | 0 | 0 | 0 | 0 | 0 | 0 | <90 | 50-75 | 70 |
| 96 | 0 | 0 | 0 | 0 | 0 | 0 | <90 | 50-75 | 70 |
| 97 | 0 | 25 | 20 | 0 | 0 | 0 | 50 | 50 | 50 |
| 98 | 0 | 0 | 0 | 0 | 0 | 0 | 0 | 0 | 0 |
| 99 | 0 | 0 | 0 | 0 | 0-25 | 20 | 100 | 100 | 100 |
| 100 | 0-25 | 25-50 | 25 | 0 | 0 | 0 | 100 | 100 | 100 |
| 101 | 0 | 0 | 0 | 0 | 0 | 0 | 100 | 100 | 100 |
| 102 | 0 | 0 | 0 | 0 | 0 | 0 | 0 | 0 | 0 |
| 103 | 0 | 25 | 20 | 0 | 0 | 0 | 100 | 100 | 100 |
| 104 | 0 | 0 | 0 | 25 | 25 | 25 | 50 | 25 | 50 |
